# RNA Sequencing Reveals That Both Abiotic and Biotic Stress-Responsive Genes Are Induced during Expression of Steroidal Glycoalkaloid in Potato Tuber Subjected to Light Exposure

**DOI:** 10.3390/genes10110920

**Published:** 2019-11-11

**Authors:** Weina Zhang, Cunwu Zuo, Zhongjian Chen, Yichen Kang, Shuhao Qin

**Affiliations:** 1 College of Horticulture, Gansu Agricultural University, Lanzhou 730070, China; nanana20082008@126.com (W.Z.); zuocw@gsau.edu.cn (C.Z.); yichenkang@sina.com (Y.K.); 2 Agro-Biological Gene Research Center, Guangdong Academy of Agricultural Sciences, Guangzhou 510640, China; chenzhongjian@agrogene.ac.cn

**Keywords:** potato, steroidal glycoalkaloids, Mapman, co-expression, disease resistance

## Abstract

Steroidal glycoalkaloids (SGAs), which are widely produced by potato, even in other *Solanaceae* plants, are a class of potentially toxic compounds, but are beneficial to host resistance. However, changes of the other metabolic process along with SGA accumulation are still poorly understood and researched. Based on RNA sequencing (RNA-seq) and bioinformatics analysis, the global gene expression profiles of potato variety Helan 15 (Favorita) was investigated at four-time points during light exposure. The data was further verified by using quantitative Real-time PCR (qRT-PCR). When compared to the control group, 1288, 1592, 1737, and 1870 differentially expressed genes (DEGs) were detected at 6 h, 24 h, 48 h, and 8 d, respectively. The results of both RNAseq and qRT-PCR showed that SGA biosynthetic genes were up-regulated in the potato tuber under light exposure. Functional enrichment analysis revealed that genes related to PS light reaction and Protein degradation were significantly enriched in most time points of light exposure. Additionally, enriched Bins included Receptor kinases, Secondary metabolic process in flavonoids, Abiotic stress, and Biotic stress in the early stage of light exposure, but PS Calvin cycle, RNA regulation of transcription, and UDP glucosyl and glucoronyl transferases in the later stage. Most of the DEGs involved in PS light reaction and Abiotic stress were up-regulated at all four time points, whereas DEGs that participated in biotic stresses were mainly up-regulated at the later stage (48 h and 8 d). *Cis*-element prediction and co-expression assay were used to confirm the expressional correlation between genes that are responsible for SGA biosynthesis and disease resistance. In conclusion, the expressions of genes involved in PS light reaction, Abiotic stress, and Biotic stress were obviously aroused during the accumulation of SGAs induced by light exposure. Moreover, an increased defense response might contribute to the potato resistance to the infection by phytopathogenic microorganisms.

## 1. Introduction

Potato (*Solanum tuberosum* L.) is one of the most important staple crops for direct and processed consumption in many countries around the world. It serves as major, inexpensive low-fat food source, providing carbohydrates and containing high-quality proteins as well as antioxidative polyphenols, vitamins, and minerals [1]. Potato tubers possess small quantities of naturally occurring steroidal glycoalkaloids (SGAs), which are explained as stress metabolites or phytoalexins for protecting the potato plant from insect pests and herbivores [2]. However, SGAs can be hazardous for human health when the level of total SGA is up to 200 mg kg^−1^ fresh weight of unpeeled raw potato tuber [3]. It can induce mild symptoms of glycoalkaloid toxicity, including headache, nausea, and diarrhea, but more severe and even fatal poisonings have been reported [4]. The SGA content is correlated with the resistance of potato against pests, whereas the reduction of potato SGAs for the sake of food safety might weaken pathogen resistance [5]. Furthermore, several studies have demonstrated that resistance to the beetles (*Leptinotarsa decemlineata*) is associated with potato SGAs, and a higher level of SGAs can impart strong resistance [2]. However, previous reports found that SGAs were not responsible for the resistance to late blight of hybrids [6,7]. These investigations are only limited to the relationship between SGA content and potato resistance; hence, an increased understanding of the relationship of SGA biosynthetic pathway and potato molecular defense responses is of broad interest.

SGAs are widely spread throughout the family *Solanaceae* [8,9]. A lower level of SGAs was produced in the tubers, while the highest concentration is in some tissue types, such as flowers, unripe berries, young leaves, and sprouts, which are described as bitter tasting, burning, scratchy, or acrid [2,10,11]. In cultivated potatoes, over 95% of the SGAs are *α-*solanine and *α-*chaconine, which are especially present in sprouts, the green peel of tubers, and other aerial parts [2,10]. The ability of potato to accumulate SGAs varies between cultivars, and remarkably influenced by the environment as well, such as wounding, mechanical injury, light exposure, nitrogen fertilization, inappropriate storage, or extreme temperature [12,13,14]. Some research showed that the increase of SGA concentration is often associated with greening that is exposed to light, but greening and SGA accumulation was under separate genetic control [15,16]. However, no previous study has reported the transcriptome dynamics of potato tubers that are involved in the process until now.

SGAs are derived from the isoprenoid pathway, and cholesterol (CHR) has been identified as a common precursor [17,18]. In potato and tobacco (*Nicotiana tabacum*), CHR is a major sterol, which accounts for between 5% and 20% of the 4-desmethyl sterols [19,20]. In the biosynthesis pathway, the formation of high levels of the total SGA was associated with the transcript level of HMG-CoA reductase 1 and 2 (*HMG1* and *HMG2*), potato vetispiradiene synthase 1 (*PVS* 1), and potato squalene synthase 1 (*PSS* 1). However, the ratio between solanine and chaconine appears to be associated with the relative expression level of solanidine galactosyltransferase 1 (*SGT* 1) and solanidine galactosyltransferase 2 (*SGT* 2) and 3 (*SGT* 3) [21,22].

Light plays a vital role in the process of photosynthesis. Under high light, the global gene expression patterns were identified in moso bamboo (*Phyllostachys edulis*) based on RNA-Seq data, in which 47 gene annotations that were closely related to photosynthesis were refined. The pathway of reactive oxygen species (ROS), as well as identified ROS-scavenging genes and transcription factors in the regulation of photosynthetic and related metabolisms, were detected [23]. Jung et al. (2018) reported that change in the transcriptome of Ginseng, in which most of the up-regulated transcripts were related to stress responses, while many down-regulated transcripts were related to cellular metabolic processes [24]. Under blue light induction, genes that have a significant effect on processes of circadian rhythm, flavonoid biosynthesis, photoreactivation, and photomorphogenesis were identified [25]. However, the SGA biosynthesis and plant disease resistance gene expression profiles in response to light exposure were elusive in potato. We first applied the RNA sequencing (RNA-Seq) approach to investigate the dynamic changes of potato transcriptome during light exposure, and the expression patterns of seven SGA biosynthetic genes were confirmed using the quantitative Real-Time PCR (qRT-PCR). Our analysis revealed numerous differentially expressed genes (DEGs) and ontology bins that are involved in both abiotic and biotic stress, and, during the process of SGA gene expression, plant resistance genes were activated. The relationships were further detected by using *cis*-elements prediction and co-expression network construction. Our study shows an important implication for potato resistance breeding and it may contribute to a better scientific understanding of SGAs and defense regulation in plants.

## 2. Methods

### 2.1. Biological Materials and Treatment

**Figure 1:**
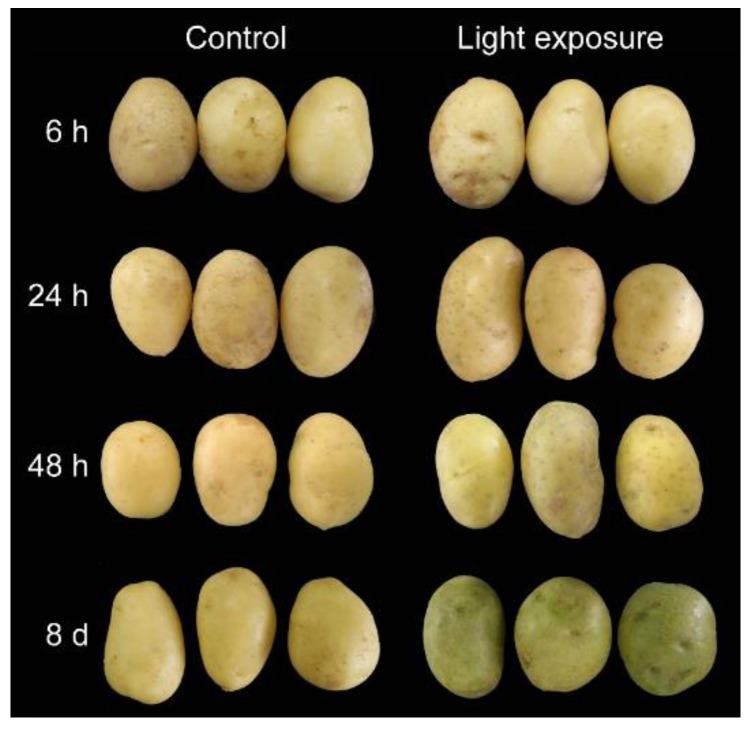
General appearance of potato tubers subjected to light exposure. Tubers were exposed to constant white fluorescent light in a growth cabinet for the time points indicated (6 h, 24 h, 48 h, and 8 d). Additionally, for the control, potatoes were placed in the dark in the same cabinet.

The potato cultivar ‘Helan 15’ (Favorita cultivar, light yellow, and smooth skin, with small and shallow bud eyes) (Figure 1) was used for light exposure, which was obtained from Dingxi Academy of Agricultural Sciences (Gansu, China). Helan 15 was introduced by Crop Detoxification Technology Development Center of Minhe County (Qinghai, China) from the Institute of Plant Protection, Tianjin Academy of Agricultural Sciences (Tianjin, China), and its deposition number is Qingshenshu 2007001 (NCBI BioSample ID: SAMN11865697). The tubers with similar weight (72 ± 5 g) and free of visible damage were placed in a growth cabinet at 25 °C under constant white fluorescent light (3000 Lux) for 6 h, 24 h, 48 h, and 8 d, defined as T1, T2, T3, and T4, respectively. For control samples, the tubers were kept in the dark at the same time points and defined as C1, C2, C3, and C4, respectively (Figure 1). For each sample, tissues with 1.5 mm thick in the surface of three tubers with the same treatment were harvested, immediately frozen in liquid nitrogen, and then stored at −80 °C. Each treatment included three biological replicates.

### 2.2. RNA Extraction, RNA-Seq Library Construction and Sequencing

For all the samples, total RNAs were extracted while using the Plant RNAout kit (160906-50, Tiandz Inc., Beijing, China) according to the manufacturer’s instructions. Extracted RNA was quantified and qualified by using a NanoDrop2000 Spectrophotometer (Thermo Scientific, MA, Waltham, United States) and an Agilent 2100 Bioanalyzer (Agilent Technologies, Palo Alto, CA, USA), respectively. Poly(A) mRNA was purified by using oligo(dT) magnetic beads and then digested into short fragments (approximately 200 bp). The RNA-Seq library preparation method were performed according to our previous method that was described by Zuo et al. (2017) [26] and it included three replicates for each treatment. The amplified fragments were sequenced while using an Illumina HiSeq^TM^ 2000. The produced reads were paired-end 2 × 150 bp. All the data pertaining to the present study has been included in the tables/figures of the manuscript and the raw data of RNA sequencing has been deposited at the Sequence Read Archive (SRA) database of NCBI (SRS4823473). The authors are pleased to share the data upon request.

### 2.3. Analysis of Sequencing Data

RNA-Seq bioinformatics analysis was used to analyze the transcriptome raw sequences. The original images were converted into sequence data by base calling to provide the raw reads. Subsequently, dirty reads were removed before data analysis to acquire the clean reads. The clean reads were mapped to the genome sequence (v4.04) of *S. tuberosum* [27]. Gene expression levels were quantified by using the RSEM v1.2.20 with BAM file alignment by Bowtie2 v2.2.3 (https://doi.org/10.5281/ZENODO.572271) [28] and default RSEM parameters (RNA-Seq by Expectation Maximization) [29]. Gene identification (gene ID), length, log2 ratio, and false discovery rate (FDR) were obtained. An FDR value ≤0.01 and an absolute value of log2 ratio ≥2 were both used as threshold to judge the significant differences in gene expression. After that, the expression data of DEGs at different time points were submitted to software Mapman [30]. Sixteen most enriched ‘Bins’ with a level equal to ‘2’ from each treatment were extracted. The expressional patterns of DEGs involved in enriched Bins were extracted by gene accession. Heat maps were constructed by using a Multiple Array Viewer software MeV 4.9.0 (http://www.tm4.org/mev.html).

### 2.4. Cis-Element Prediction and Weighted Gene Co-Expression Network Analysis (WGCNA)

The promoter sequences (1.5 kb upstream regions) of target genes were retrieved from the potato genome sequence v4.04 [27]. *Cis*-elements were predicted by using database PlantCARE (http://bioinformatics.psb.ugent.be/webtools/plantcare/html/ [31]). Correlation among all of the samples was detected by the outlier analysis. Disease resistant genes were predicted while using blastp v2.6.0+ (-max_hsps 1-evalue 1 × 10^−10^- qcov_hsp_perc 50) with R-genes from PRGDB (http://prgdb.crg.eu/wiki). After that, co-expression networks of genes that are responsible for SGA biosynthesis and disease resistance were constructed with WGCNA [32]. Module Eigengenes (ME) values were used to estimate the associations between the modules and samples.

### 2.5. Quantitative Real-Time PCR

Quantitative Real-Time PCR (qRT-PCR) was used to further validate twelve DEGs that were involved in enriched ‘Bins’. For cDNA synthesis, 500 ng of total RNA was transcribed to cDNA by using the PrimeScript™ RT reagent Kit with gDNA Eraser (Code No. RR047A, TAKARA Biotechnology Co., Ltd., Dalian, China). Primer design was performed by using the online software program Primer 3 (http://primer3.ut.ee/). The primer sequences are shown in Additional file S1: Table S1. PCR conditions were 95 °C for 15 min., followed by 40 cycles of 95 °C for 10 s, and annealing/extension at 60 °C for 30 s. The melting curve was determined for each sample. Relative gene expression was calculated while using the cycle threshold (Ct)2^−ΔΔCt^ method, as described by Zuo et al. (2017) and Livak and Schmittgen (2001) [26,33]. Data from qRT-PCR analysis were expressed as means ±SD of three independent replicates.

## 3. Results

### 3.1. Potato Tubers with a Difference in Phenotypes

Time-dependent exposure experiments were performed to investigate the change of potato under light. Light exposure followed by phenotypic observation showed that the distinct green color was dependent on the duration of light exposure (Figure 1). A greener color became obvious by the eye when the tubers were exposed to light for 48 h, and when the light exposure reached eight days, the potato skin turned the greenest. Potatoes in the dark had no obvious change in color. Except skin color, no significant difference was found between treated and non-treated tubers.

### 3.2. Quality of Data and Differentially Expressed Genes 

**Figure 2:**
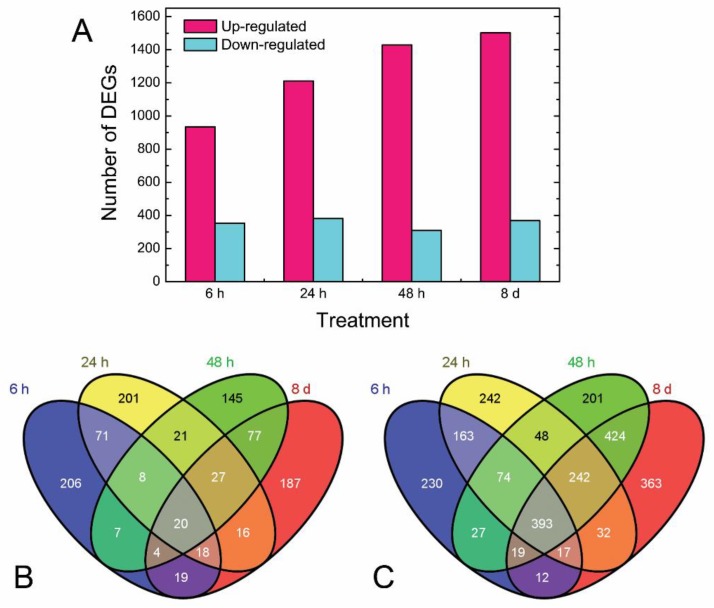
Comparative analysis of differentially expressed genes (DEGs) at 6 h, 24 h, 48 h, and 8 d. (**A**): Number of DEGs from samples with light exposure compared with that in dark at the same time point. (**B**,**C**): Venn diagram summarizing the down-regulated (**B**) and up-regulated (**C**) DEGs detected among four time points (6 h, 24 h, 48 h, and 8 d). Common and specific genes to each time point treatment are represented in the photograph.

A total of 24 RNA samples, which were collected from three biological replicates of with or without light exposured tubers at four time points (6 h, 24 h, 48 h, and 8 d) were subjected to RNA-seq. The results are shown in Table 1. After filtering the raw sequence reads, more than 40 million clean reads were obtained from each sample. Genome and gene mapping ratio ranged from 74.38% to 80.43% and from 61.32% to 65.58%, respectively (Additional file S2: Table S2), which indicated that the sequences were appropriate for further analysis. From each sample, we detected approximately twenty thousand expressed genes, which indicated that our data could be expected to identify most of the genes expressed under each condition. When compared to samples in the control group at the same time point, 1288, 1592, 1737, and 1870 differentially expressed genes (DEGs) were found from 6 h, 24 h, 48 h, and 8 d, respectively (Figure 2A; FDR ≤ 0.01 and log2 ratio ≥ 2). Approximately 75% of DEGs were up-regulated, which is an indication of metabolic processes that were aroused in potato during light exposure. Overlapping of the down-regulated DEGs among the samples from four time points showed that most of these were expressed in each sample. However, a great number of up-regulated DEGs (393) commonly existed in the four samples. The detailed expression data of all DEGs are presented at Additional file S3: Table S3.

### 3.3. Expression Patterns of the Steroidal Glycoalkaloid (SGA)-Related Genes

**Figure 3:**
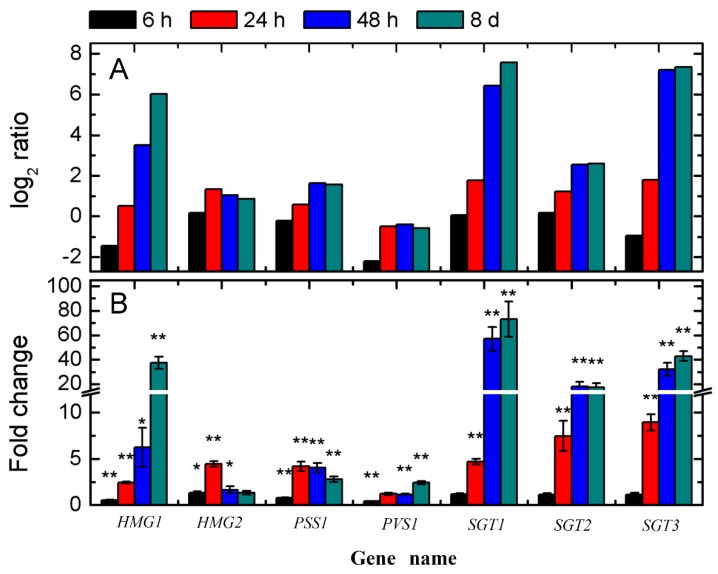
Temporal expression of SGA-related genes in potato tuber subjected to light exposure. (**A**): Expression data of seven steroidal glycoalkaloid (SGA)-related genes retrieved from RNA-seq data. (**B**): qRT-PCR detection for expression patterns of SGA-related genes. For the qRT-PCR data, the means ± SD for the three replicates are represented. Error bars represent the range of relative expression (qPCR fold change) calculated by 2^−(∆∆Ct^^±SD)^ (*n* = 3). The asterisks indicate a significant difference as compared with the group in the dark at each time point (* *p* < 0.05 and ** *p* < 0.01).

A previous study has demonstrated that *HMG1* and *HMG2* were the primary metabolism genes, while *SGT1*, *SGT2,* and *SGT3* were the secondary metabolism genes, which were demonstrated to be directly involved in SGA biosynthesis, and the expression of *PSS1* and *PVS1* were associated with the accumulation of SGAs [21,22]. The overexpression of *HMG1* in potato plant increased SGA content in potato leaves [22]. Here, we extracted expression patterns of seven SGA-related genes from the current RNA-seq data (Figure 3A). Four of these genes were obviously up-regulated, including *HMG1* (PGSC0003DMP400024174), *SGT1* (PGSC0003DMP400020829), *SGT*2 (PGSC0003DMP400030574), and *SGT*3 (PGSC0003DMP400020813). After light exposure, the log_2_ ratio values of *HMG*1, *SGT*1, *SGT*2, and *SGT*3 were recorded as 3.51, 6.43, 2.56, and 7.21 at 48 h, and as 6.02, 7.58, 2.59, and 7.34 at 8 d, respectively. qRT-PCR detection further showed similar trends for each gene between two methods (Figure 3B). The results presented herein showed that light could induce the expression change of these genes that are involved in the generation of SGAs.

### 3.4. Overview of Mapman Analysis

**Figure 4:**
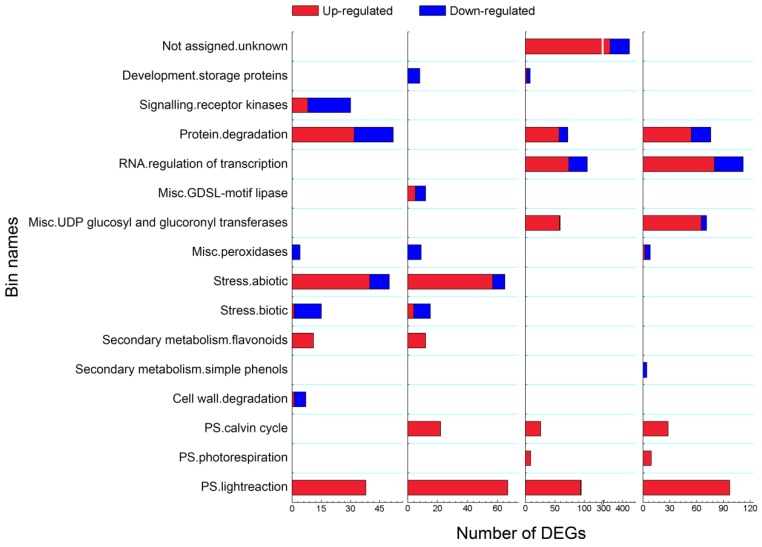
Functional classification analysis of differentially expressed genes (DEGs) at 6 h, 24 h, 48 h, and 8 d of light exposure. The sixteen most enriched Bins as a level equal to ‘2′ in each sample are listed. The genes related to PS (Photosynthetic system), biotic and abiotic stress, and some other progresses involved were shown. Gene number of biological processes in up-regulated (red) and down-regulated (blue) are represented. Abbreviations are as follows: PS: Photosystem.

The most enriched Bins as a level equal to ‘2’ in each sample are displayed in Figure 4. In total, sixteen Bins were enriched at least in one sample. The enriched Bins PS light reaction, PS Calvin cycle, Peroxidase, and Protein Degradation were commonly detected from at least three time points. Enriched Bins Receptor kinases and Cell wall degradation were only found from the first time point of light exposure, and most DEGs showed a down-regulation. Bins Abiotic stress, Biotic stress, and Secondary metabolic process in flavonoids were detected at both 6 h and 24 h. Additionally, RNA regulation of transcription, UDP glucosyl and glucoronyl transferases, and PS photorespiration were commonly enriched at 48 h and 8 d. The above results indicated that photosynthesis and several other metabolic pathways of potato tuber were aroused by light exposure.

All of the DEGs that were involved in PS light reaction, PS Calvin cycle, PS photorespiration, and Secondary metabolic process in flavonoids displayed an up-regulation pattern, while DEGs that belonged to Biotic stress, Peroxidase, Receptor kinases, Receptor like kinase, Storage protein, and Cell wall degradation and Simple phenols were mainly down-regulated. For all of the other enriched Bins, most involved DEGs showed an up-regulated pattern. We noticed that expression of DEGs involved in both Biotic stress and Abiotic stress was influenced by light exposure at 6 h and 24 h.

### 3.5. DEGs Involved in Abiotic Stress Were Differentially Expressed in Response to Light Exposure

**Figure 5:**
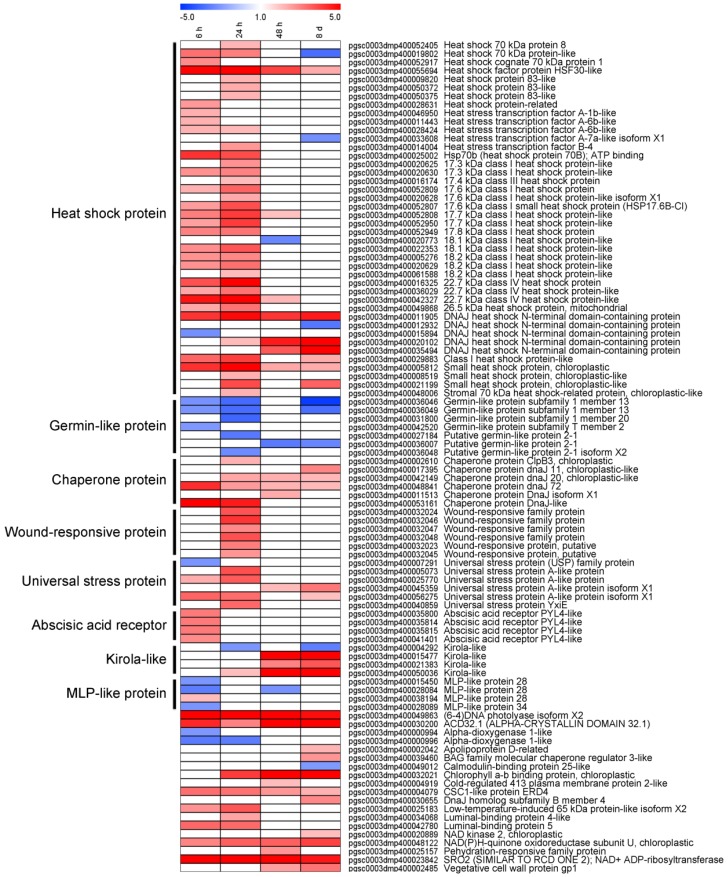
Heat map showed transcriptional profiles of abiotic-related DEGs, which included heat shock proteins, germin-like proteins, chaperone proteins, wound-responsive proteins, universal stress proteins, Abscisic acid receptors, Kirola-like proteins, and MLP-like proteins. Bins in red are significantly up-regulated; bins in blue are significantly down-regulated.

A comprehensive illustration of DEGs that are related to Abiotic stress in potato tuber, which was influenced by light exposure, is presented in Figure 5. In total, 98 DEGs were differentially expressed at least in one time point, 42 of these encoding heat shock protein, seven germin-like protein subfamily, six chaperone protein, universal stress protein and wound-responsive protein, and four abscisic acid receptor, kirola-like protein, and MLP-like protein, etc. We noticed that the up-regulated DEGs mainly encoded heat shock protein, chaperone protein, wound-responsive protein, universal stress protein, abscisic acid receptor, and kirola like protein, whereas the down-regulated DEGs encoded germin-like protein and MLP-like protein. These results indicated that a multiple abiotic response in potato tuber had been activated during light exposure, especially in the early stage.

### 3.6. DEGs Involved in Biotic Stress Were Differentially Expressed in Response to Light Exposure

**Figure 6:**
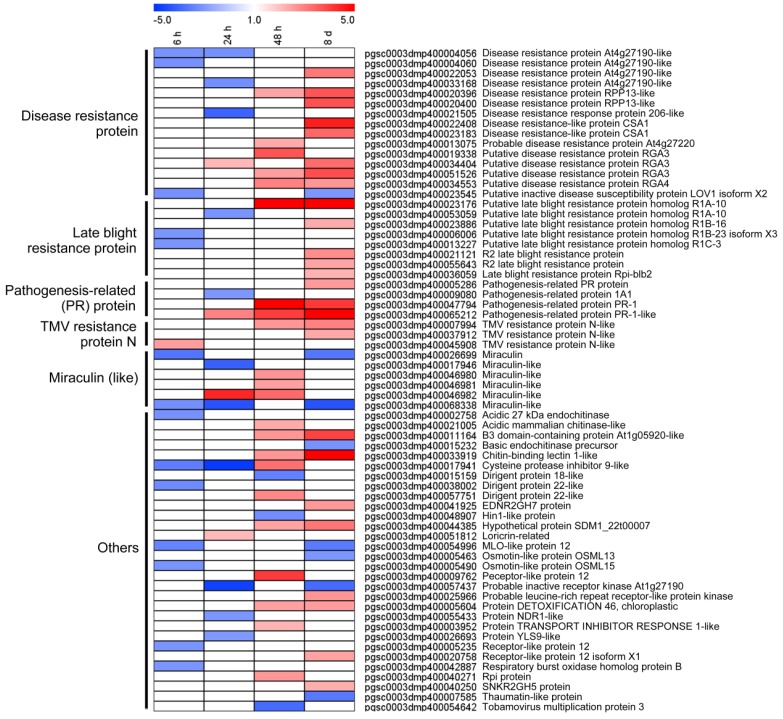
Heat map showed transcriptional profiles of biotic-related DEGs, which included disease resistance proteins, late blight resistance proteins, PR (pathogenesis-related) proteins and so on. Bins in red are transcribed at higher levels; bins in blue are transcribed at lower levels. Results indicate that stronger activation of biotic-related bins occurs in the later stage.

DEGs expressional patterns that were involved in enriched Bin Biotic stress were investigated. As shown in Figure 6, we identified six representative clusters, comprising a total of 15 DEGs, which were found from potato tuber in the early stage of light exposure (both 6 h and 24 h), most of which were down-regulated (93.3% and 73.3% at 6 h and 24 h, respectively). Very importantly, we discovered that DEGs in the Bins displayed stronger transcription at the later stage (48 h and 8 d). It is especially relevant to note that many of the bins associate with plant disease, including disease resistance protein, late blight resistance protein, pathogenesis-relate (PR) protein, and tobacco mosaic virus (TMV) resistance protein N, and most of these genes (53.3% disease resistance protein, 62.5% late blight resistance protein, 75% PR protein, 66.7% TMV resistance protein) were highly expressed at 8 d. These results suggested that the expression of DEGs that were involved in ‘biotic stress’ were only up-regulated in the later stage (48 h and 8 d) of light exposure.

### 3.7. Cis-Elements in the Promoter Region of SGA-Biosynthetic and Disease-Resistant Genes

**Figure 7:**
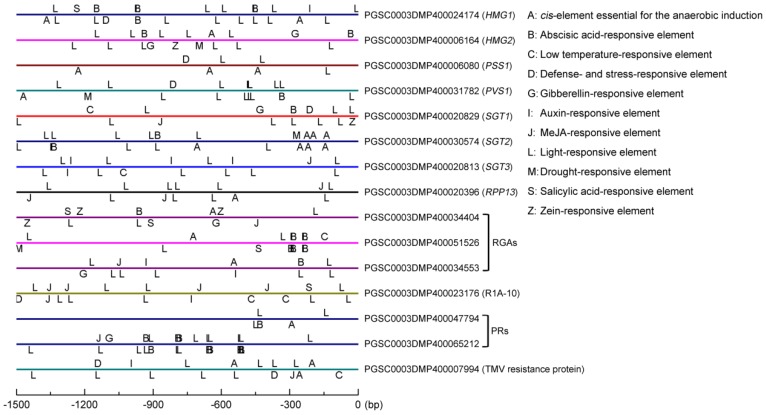
Distribution of *cis*-elements in the promoter region of seven SGA biosynthetic genes (*HMG1*, *HMG2*, *PSS1*, *PVS1*, *SGT1*, *SGT2,* and *SGT3*) and eight disease resistant genes. For disease resistant genes, one of these encodes the disease resistant protein RPP13 (PGSC0003DMP400020396), three for disease resistant protein RGAs (PGSC0003DMP400034404, PGSC0003DMP400051526 and PGSC0003DMP400034553), one for late blight resistance protein R1A-10 (PGSC0003DMP400023176), and one for TMV resistance protein (PGSC0003DMP400007994).

Light-responsive elements (L) were ubiquitous and relatively abundant on the promoters of SGA-biosynthetic and disease-resistant genes. Furthermore, we also found many hormones responsive elements, such as B (abscisic acid (ABA)), G (gibberellins (GA)), I (Auxin (IAA)), J (methyl jasmonate (MeJA)), and S (salicylic acid (SA)), and adversity (C: low temperature, D: drought, M: defense) responsive elements from several SGA biosynthetic genes and disease-resistant genes (Figure 7). For example, four ABA-responsive elements were detected from *HMG1* (PGSC0003DMP400024174), five and eight from gene encoding disease-resistant protein RGA (PGSC0003DMP400051526) and PR (pathogenesis-related) protein (PGSC0003DMP400065212), respectively. Additionally, defense- and stress-responsive elements were discovered from the promoter regions of four SGA biosynthetic genes (*HMG1*, *PSS1*, *PVS1,* and *SGT1*) and two disease-resistant genes (PGSC0003DMP400023176 and PGSC0003DMP400007994). The above results indicated that both SGA biosynthetic and disease-resistant genes might co-respond to signals from light, hormone, and stress, and their functions need to be further verified.

### 3.8. Verification of DEGs Using qRT-PCR

**Figure 8:**
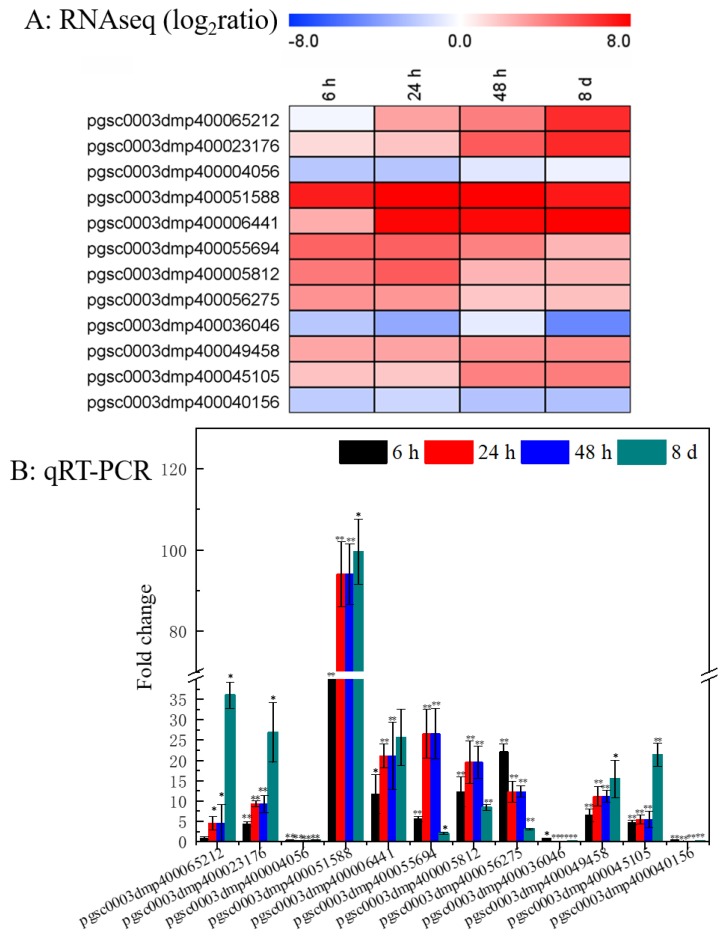
Comparison of RNA-Seq and qRT-PCR analyses for the gene’s expression validation. (**A**): Heatmaps showed transcriptional profiles of candidate DEGs from the enriched Bins. Red indicates genes that are up-regulated, and blue indicates genes that are down-regulated. (**B**): Relative gene expression determined by qRT-PCR. Gene expression was presented as mean value of the light treated tubers as compared to the dark treated tubers for each time point. Error bars represent the range of relative expression (qPCR fold change) calculated by 2^−(∆∆Ct^^±SD)^ (*n* = 3). The asterisks indicate a significant difference compared with the group in dark at each time point (* *p* < 0.05 and ** *p* < 0.01).

The expression patterns of 12 representative DEGs involved in enriched Bins were confirmed using qRT-PCR assay to verify the reliability of the RNA-seq data (Figure 8B). Three biotic responsive genes encoding PR protein (pgsc0003dmp400065212), late blight resistance protein (pgsc0003dmp400023176), and disease resistance protein (pgsc0003dmp400004056) were checked by qRT-PCR assay. Additionally, two genes that were involved in flavonoid metabolism (pgsc0003dmp400051588 and pgsc0003dmp400006441), five in abiotic stresses (pgsc0003dmp400055694, pgsc0003dmp400005812, pgsc0003dmp400056275, pgsc0003dmp400036046 and pgsc0003dmp400049458), and two receptor-like kinase genes (pgsc0003dmp400045105 and pgsc0003dmp400040156) were also detected. Similar changes in all detected genes were observed between RNA-seq (Figure 8A) and qRT-PCR assays (Figure 8B), as indicated the accuracy of the RNA-seq data.

### 3.9. Co-Expressional Networks of Genes between SGA and Disease Resistance

**Figure 9:**
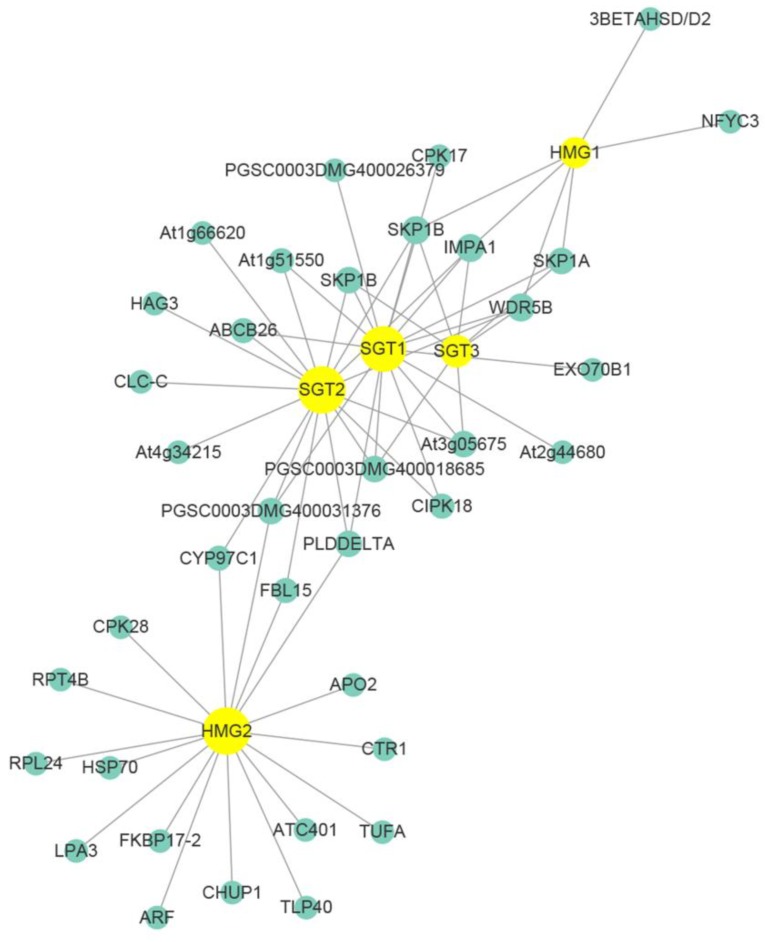
Co-expressional networks of genes responsible for SGA biosynthesis and disease resistance. In the photograph, the SGAs biosynthesis genes were marked in yellow, and the disease resistance genes were marked in green.

WGCNA is designed for constructing co-expression networks from microarray-based expression data and it not only considers the co-expression patterns between two genes, but also the overlap of neighboring genes. While using recently available R-gene database (http://prgdb.crg.eu/wiki), we constructed a gene co-expression network between SGA biosynthesis and disease resistance using WGCNA. Four distinct transcription modules were identified from the transcriptome data (Supplementary Figure S1). Different samples were correlated with four distinct modules, in which module ‘blue’ (MEblue) and module ‘turquoise’ (MEturquoise) were highly correlated with the samples (Supplementary Figure S2). After filtering, the MEturquoise module, including five SGAs biosynthetic genes, was used for constructing the co-expression network. As shown in Figure 9, the SGA biosynthesis genes (yellow marked in Figure 9) were correlated with the disease resistance genes (green marked in Figure 9) in different patterns (Figure 9). Some disease-resistant genes, such as IMPA1 (PGSC0003DMG400014989), SPK1B (PGSC0003DMG400006184), and WDR5B (PGSC0003DMG400019361), were co-expressed to multiple SGA biosynthetic genes (HMG1, SGT1, SGT2, and SGT3), while CIPK18 (PGSC0003DMG400020550) and At1g51550 (PGSC0003DMG400026513) were co-expressed to SGT1 and SGT2 (Figure 9 and Additional file S4: Table S4). Above all, we suggest that potato SGA biosynthesis positively regulates its disease resistance.

## 4. Discussion

In the current study, we investigated the gene expression profiles of the potato tuber between SGAs and biotic stress that is induced by light exposure. We confirmed the significant up-regulation of all seven SGA biosynthesis genes, indicating gene expression induction of SGA along with light exposure (Figure 2 and Figure 3; [34]). Subsequently, we also found the expression of genes correlated with plant disease resistance were activated in the process. *Cis*-element prediction and co-expression networks further verified the relationship. To our knowledge, this is the first investigation regarding the correlation of gene expression between the stress response and SGA accumulation induced by light exposure.

Light is an important factor for plant development and it has crucial effects on the growth, production, and quality of potatoes [35]. Several authors have demonstrated that light could also increase the SGAs concentration to twice or three times when compared with the initial levels in potato, which occurred either in the field, at harvest, or during storage [3,9,36]. Gull and Isenberg (1960) [37] and De Maine et al. (1998) [38] found that the content of the observed chlorophyll formation associated with greening and of solanine subjected to light exposure developed independently. In our present study, we found that during the light exposure, a green color in tuber peel became obviously by the eye (Figure 1), which was well in accordance with the previous research [3]. Transcriptome changes were compared to identify the genes underlying the light exposure (Figure 2). When compared with the sample in darkness at the same time point, about 75% of DEGs were up-regulated (Figure 2C), and the SGA biosynthesis-associated genes, especially *SGT1*, *SGT2,* and *SGT3* (24 h, 48h, and 8 d, *p* < 0.01) were up-regulated in the process by the transcript profiling and qRT-PCR (Figure 3). During the up-regulation of photosynthesis and SGA biosynthesis-associated genes, many DEGs that are involved in stresses (biotic and abiotic) response and flavonoid metabolism were discovered (Figure 4). For abiotic stress, most up-regulated DEGs encoded heat shock protein, chaperone protein, wound-responsive protein, universal stress protein, and abscisic acid receptor (Figure 5). SGAs are toxic compounds to insects, bacteria, and animals, but they have been suggested to have defensive functions for potato [39,40,41,42]. Several mechanisms of SGA toxicity are suggested, such as the disruption of the membrane fluidity and the inhibition of cholinesterase activity [42,43,44]. Although little investigation was involved in the correlation of gene expression between light-induced SGA biosynthesis pathway and potato defense responses, several stress treatments, such as wounding and light exposure, increased potato tuber SGAs level indicated that SGA biosynthetic pathway is probably associated with abiotic defense responses [45], which was consistent with our results (Figure 5). Multiple abiotic stress-responsive proteins can be induced by one stress factor. For instance, the rapid accumulation of wound response proteins was identified from both light and water stress treated plant tissues [46]. Additionally, heat shock protein contributes to plant tolerance to multiple stress, such as heat, drought, etc. [47]. Although the current investigation suggests a probable relationship that is based on these experiments, further confirmation for the correlation between SGA biosynthesis and abiotic stress responses are needed in the future.

Additionally, most of the DEGs that are involved in biotic stress encoding disease protein, late blight resistance protein, PR protein, and TMV resistance protein N were down-regulated at 6 h and 24 h, but they showed an opposite trend at 48 h and 8 d (Figure 6). For a long time, opinions have existed for regarding relationship between SGA biosynthesis and disease resistance. In many cases, potato SGA accumulation was correlated with its resistance to *Fusarium solani* var. *coeruleum*, *Fusarium sulphureum* [16], *Phytophthora infestans* [6,7], and *Clavibactermic higanensis* ssp. *Sepedonicus* [48]. On the contrary, infection of *P. infestans* and *Rhizoctonia solani* showed no obvious influences on the SGA level in potato tuber [49], because these phytopathogenic microorganisms could overcome SGA toxicity and directly resist pathogen infection [42]. However, from our results, the expression of disease-resistant genes was reduced or it did not significantly change in the early stage of light exposure, but it was strongly induced in the later stage, where a common signal-transduction pathway might be activated at the later stage, which leads to PR-protein and other biotic stress protein accumulation. For a resistant wild potato *S. arcanum* to the early blight, the important roles of SGAs biosynthetic genes on its resistance have been verified by both non-targeted metabolomic and functional genomics analysis [50]. The results indicated that the key genes involved in SGA metabolism were positively regulated for potato disease resistance, which was consistent with our results (Figure 6).

PlantCARE was used to predict the upstream promoter elements of genes (upstream 1500-0 bp of the gene) and analyze the number of *cis*-elements related to hormones, adversity, and circadian rhythm to further explore the function of the candidate genes [51]. The results showed that, beside elements in response to light, a great number of hormone (ABA, GA, IAA, SA, ect.) and defense responsive elements were also detected (Figure 7). To our knowledge, the plant hormone plays crucial roles in plant resistant responses against pathogenic bacterial and fungal attacking [52,53]. SA mainly induce plant immune responses to hemibiotrophic pathogens, whereas JA to biotrophic and necrotrophic pathogens [54]. GA usually suppresses plant resistance against to necrotrophic infection, while IAA has a contribution to resistance [55]. Therefore, we suggested that SGAs biosynthesis were closely related to abiotic and biotic stress. Subsequently, the RNA-Seq data were validated via qRT-PCR (Figure 8). Additionally, WGCNA (weighted gene co-expression network analysis) has become a powerful practice for exploring gene-to-gene relationships and to uncover coordinately expressed gene modules [32,56]. In our study, DEGs were filtered and screened through multiple steps and criteria, and the networks were then constructed while using SGA biosynthetic genes and disease-resistant gene. Interestingly, we found SGA biosynthetic genes were closely related with disease-resistant genes in the context of gene co-expression networks (Figure 9), which can provide a theoretical basis for further research.

## 5. Conclusions

In this study, we proposed that stress gene expression, along with SGA biosynthesis, was induced by light exposure. In particular, the up-regulation of disease-resistant genes indicated that the response was correlated with SGA accumulation. The correlation between genes that are responsible for SGA biosynthesis and disease resistance was investigated by both *cis*-element prediction and co-expressional assay, in which IMPA1 (PGSC0003DMG400014989), SPK1B (PGSC0003DMG400006184), WDR5B (PGSC0003DMG400019361), CIPK18 (PGSC0003DMG400020550), and At1g51550 (PGSC0003DMG400026513) were the candidate genes for further functional study. Besides the toxicity of SGAs to phytopathogenic microorganisms, the aroused expression of disease-resistant genes during light-induced accumulation of SGAs also contributes to potato disease resistance. While our study provides some insight into the relationship between genes of SGAs and plant resistance, more efforts are required to validate and extend our findings. A critical functional experiment would be carried out to verify whether the observed patterns hold true.

## Figures and Tables

**Table 1:** Summary of sequencing and mapping results.

Sample	Raw Reads	Clean Reads	Clean Data Ratio (%)	Mapped Genome (%)	Mapped Gene (%)	Expressed Gene
C1-1	42453718	40692256	95.85	75.26%	61.32%	21502
C1-2	42453868	40784058	96.07	75.65%	62.10%	21760
C1-3	42453938	40851000	96.22	77.15%	63.42%	21234
C2-1	42452090	40489950	95.38	78.06%	63.31%	21213
C2-2	42449108	40526196	95.47	77.66%	63.54%	21240
C2-3	42450246	40465220	95.32	77.99%	63.37%	21296
C3-1	42452696	40184268	94.66	79.00%	62.55%	20684
C3-2	42454638	40396030	95.15	79.99%	64.05%	20583
C3-3	42453942	40565054	95.55	78.69%	62.36%	20724
C4-1	42453418	40439256	95.26	79.97%	62.67%	20343
C4-2	42454958	40429838	95.23	80.43%	64.14%	20208
C4-3	44085796	41531086	94.21	80.20%	64.30%	20317
T1-1	42452422	40900082	96.34	78.26%	64.01%	21252
T1-2	42453514	40644482	95.74	78.82%	65.30%	21497
T1-3	42453450	40262838	94.84	74.38%	61.61%	21439
T2-1	42452342	40865858	96.26	77.43%	63.81%	21383
T2-2	42452858	40092998	94.44	74.45%	62.40%	21523
T2-3	42452510	40548506	95.51	75.35%	62.90%	21296
T3-1	42452560	40916514	96.38	78.03%	65.32%	21088
T3-2	42454240	40920104	96.39	76.46%	64.75%	20893
T3-3	42454122	40547356	95.51	75.17%	63.14%	20942
T4-1	42453850	40831056	96.18	74.59%	62.51%	20767
T4-2	42453644	40765702	96.02	75.71%	63.58%	20667
T4-3	42453510	40945040	96.45	76.61%	65.58%	20828

## Supplementary Materials

The following are available online at https://www.mdpi.com/2073-4425/10/11/920/s1. Table S1, primers used for qRT-PCR in the present study. Table S2, summary of sequencing and mapping data from four indicated time points. Table S3, the detail expression data of all DEGs comparing control samples. Table S4, genes of SGAs biosynthesis and disease resistance used for Weighted gene co-expression network analysis (WGCNA). Figure S1, weighted gene co-expression network analysis (WGCNA) of potato. Figure S2, the correlation between modules and different samples.
