# Supplementary material for: RNA Sequencing Reveals That Both Abiotic and Biotic Stress-Responsive Genes Are Induced during Expression of Steroidal Glycoalkaloid in Potato Tuber Subjected to Light Exposure
Source: Genes (Basel). 2019 Nov 11;10(11):920. doi: 10.3390/genes10110920 (PMC6896166; doi:10.3390/genes10110920)
Supplement: Supplementary file 1 [file genes-10-00920-s001.zip › supplementary files/Supplementary Figure Legend.docx]

Supplementary Figure Legend:

Supplementary Fig. 1 Weighted gene co-expression network analysis (WGCNA) of potato. Hierarchical cluster tree showing co-expression modules. The modules are constructed using RNA-data at 6 h, 24 h, 48 h, and 8 d, respectively. The leaves in the tree present individual genes, and the major branches constitute four modules labeled by different colors.

Supplementary Fig. 2 The correlation between modules and different samples. The scale bar in the right represents the coefficient. The closer the absolute value of the correlation between potato sample and module is, the stronger the correlation is. Tubers were exposed to constant white fluorescent light in a growth cabinet for 6 h, 24 h, 48 h and 8 d, defined as T1, T2, T3 and T4, respectively. For the control group, tubers were kept in the dark in the same cabinet at the same time point and defined as C1, C2, C3 and C4, respectively.
