# Supplementary figures and images for: RNA Sequencing Reveals That Both Abiotic and Biotic Stress-Responsive Genes Are Induced during Expression of Steroidal Glycoalkaloid in Potato Tuber Subjected to Light Exposure
Source: Genes (Basel). 2019 Nov 11;10(11):920. doi: 10.3390/genes10110920 (PMC6896166; doi:10.3390/genes10110920)

Module Cluster Dendrogram

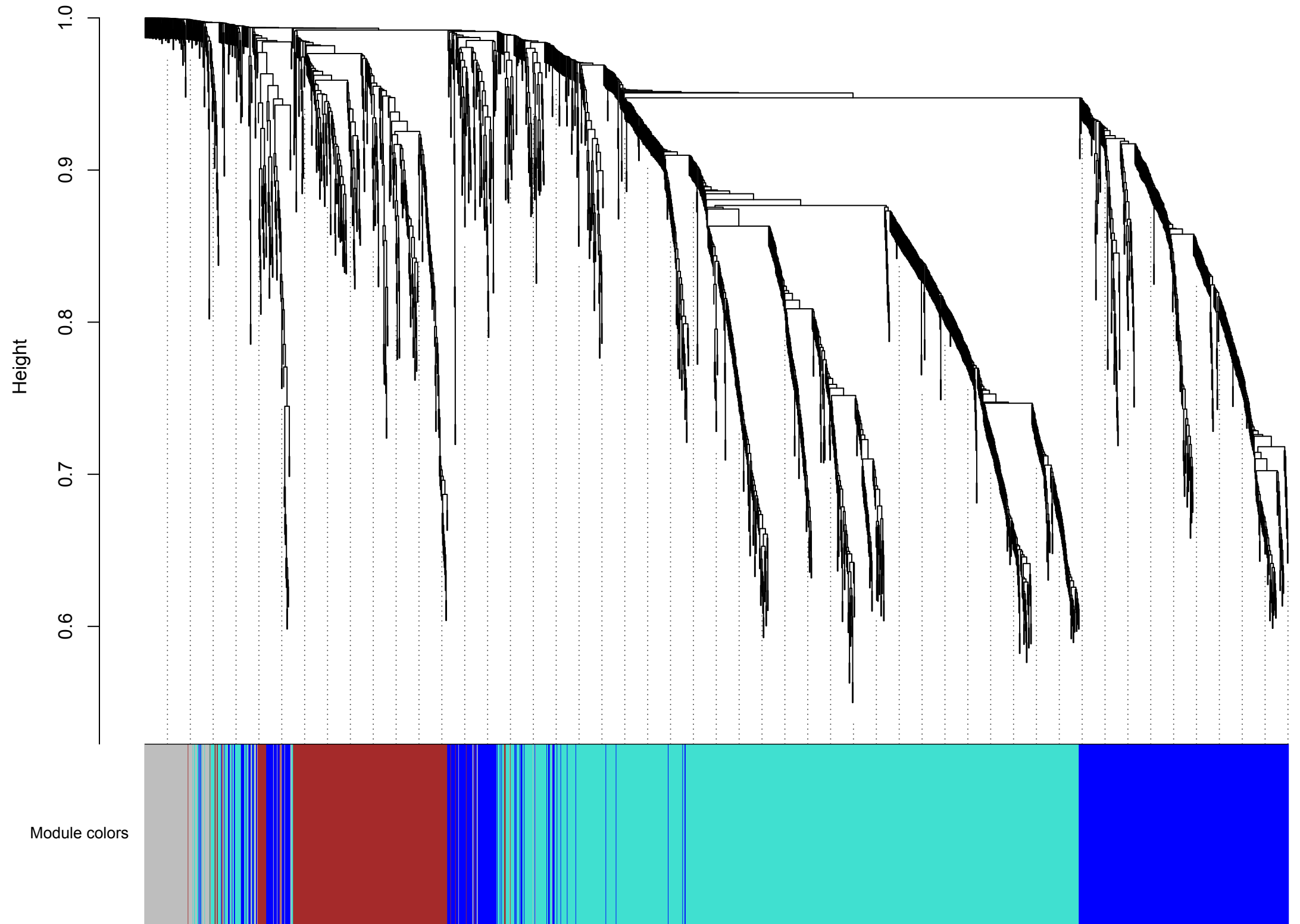

Supplement: Supplementary file 1 [file genes-10-00920-s001.zip › supplementary files/Supplementary Fig. S1 module_cluster.pdf]

# Module-sample relationships

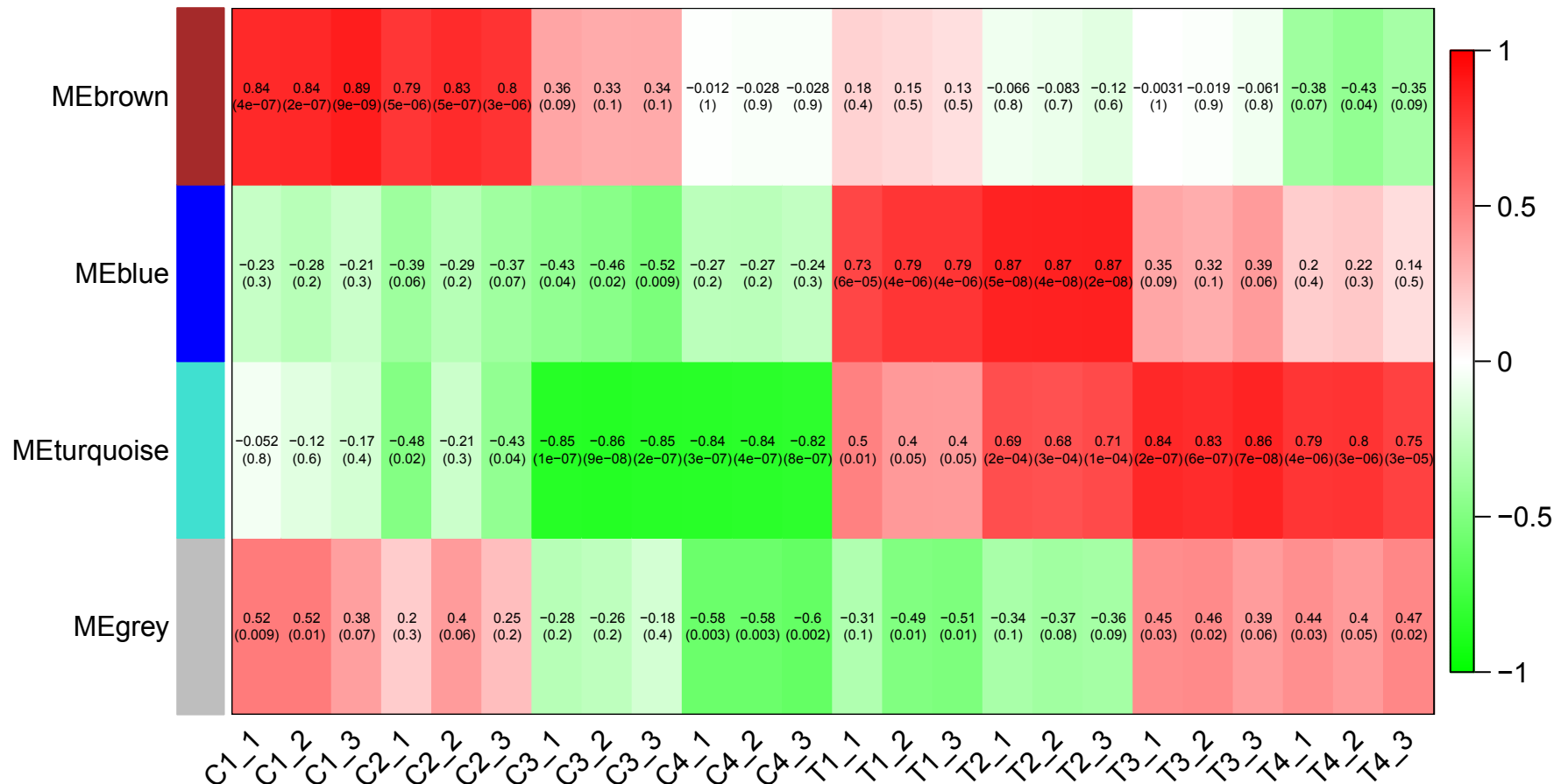

Supplement: Supplementary file 1 [file genes-10-00920-s001.zip › supplementary files/Supplementary Fig. S2 Module-sample_relationship (2).pdf]
